# Supplementary material for: The Structural Basis of the Activity Cliff in Modafinil-Based Dopamine Transporter Inhibitors
Source: Biomolecules. 2024 Jun 17;14(6):713. doi: 10.3390/biom14060713 (PMC11202288; doi:10.3390/biom14060713)
Supplement: Supplementary file 1 [file biomolecules-14-00713-s001.zip › biomolecules-3010799-supplementary.pdf]

Supporting Information

Table S1. Summary of MD simulations.

We carried out shorter simulations for the two outward-facing conditions, hDAT/RDS03-094 and hDAT/JJC8-089, because they have been equilibrated for as long as 4.5 μs in individual trajectories. However, the simulations of hDAT/RDS04-010 and hDAT/JJC8-091 transitioned from the starting outward-facing to the inward-facing state, which required long periods to equilibrated. In addition, we further extended the simulations of these two conditions to demonstrate their stability and to explore the extent of inward-facing that could be achieved.

	**number of trajectories**	**Length (ns)**
hDAT/JJC8-089	7	19,920
hDAT/JJC8-091	8	39,900
hDAT/RDS03-094	7	24,120
hDAT/RDS04-010	9	40,800

Table S2. Ligand contact frequencies of the residues interacting with RDS04-010, RDS03-094, JJC8-091, and JJC8-089 that stabilizing inward-facing or outward-facing ensembles.

In an MD frame, if the shortest heavy-atom distance between the ligand and any given residue of the protein was within 5 Å, we defined that the ligand forms a contact with this residue. The contact frequency was calculated based on the counting of the contacts per residue and normalized by the ensemble size. The contact frequencies that are above 0.5 for each of the simulated conditions are shaded in cyan. We then calculated the difference between two pairs and highlighted ∣difference value∣ ≥ 0.25. Thus, residues with light blue indicate higher contact frequencies with the sulfoxide analogs, and conversely, orange indicates the higher contact frequency with the sulfide analogs.

Residue	TM	RDS04-010	RDS03-094	JJC8-091	JJC8-089	ΔRDS*	ΔJJC#
Phe76	1m	0.83	1.00	1.00	1.00	-0.17	0.00
Ala77	1m	0.57	0.86	0.82	1.00	-0.29	-0.18
Asp79	1m	0.73	1.00	0.87	1.00	-0.27	-0.13
Ala81	1m	0.35	1.00	0.31	0.98	-0.65	-0.67
Val145	3i	0.83	0.96	0.84	0.84	-0.13	0.00
Ile148	3i	0.92	1.00	0.99	0.99	-0.08	0.00
Ser149	3i	0.98	1.00	1.00	1.00	-0.02	0.00
Val152	3m	0.97	0.76	0.99	1.00	0.21	-0.01
Gly153	3m	0.83	0.94	0.96	0.97	-0.11	-0.01
Phe154	3m	0.00	0.54	0.00	0.00	-0.54	0.00
Phe155	3m	0.00	0.52	0.01	0.00	-0.52	0.01
Tyr156	3m	0.94	1.00	1.00	1.00	-0.06	0.00
Asn157	3e	0.02	0.55	0.04	0.52	-0.53	-0.48
Phe320	6m	0.87	1.00	0.79	1.00	-0.13	-0.21
Ser321	6m	0.69	1.00	0.89	1.00	-0.31	-0.11
Leu322	6m	0.69	0.94	0.93	0.95	-0.25	-0.02
Gly323	6m	0.68	0.99	0.85	0.94	-0.31	-0.09
Phe326	6m	0.95	1.00	1.00	1.00	-0.05	0.00
Val328	6m	0.89	1.00	0.83	1.00	-0.11	-0.17
Ser422	8m	0.96	1.00	1.00	1.00	-0.04	0.00
Ala423	8m	0.81	0.97	0.96	0.94	-0.16	0.02
Gly425	8m	0.85	0.56	0.89	0.58	0.29	0.31
Gly426	8m	0.91	0.89	0.99	1.00	0.02	-0.01
Ser429	8i	0.80	0.53	0.41	0.19	0.27	0.22
Ala479	10m	0.37	0.67	0.12	0.56	-0.30	-0.44
Ile484	10i	0.84	0.87	0.74	0.91	-0.03	-0.17

*ΔRDS is the difference using RDS04-010 - RDS03-094, #ΔJJC is the difference using JJC8-091- JJC8-089.

Figure S1. The bound of JJC8-091 cannot stably interact with Asp79 resulting in the dissociation of Na2.

The evolution of the distances between the pyramidal N of the ligand and these two residues are shown in panel **B** for JJC8-091 and panel **E** for JJC8-089. The beginning binding poses of JJC8-091 and JJC8-089 are shown in **Fig. S1A** and **D** respectively, and the representative snapshots of the binding poses of JJC8-091 and JJC8-089 are shown in **Fig. S2C** and **F** respectively.

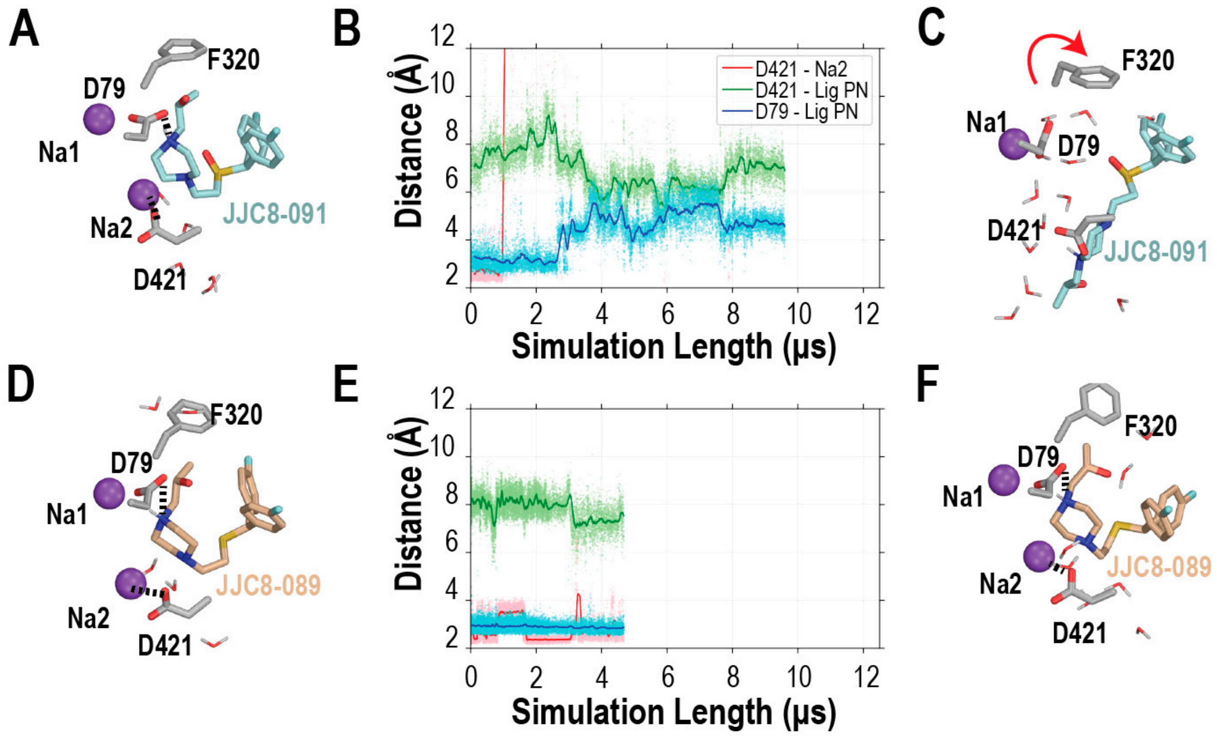

Figure S2. JJC8-091 and JJC8-089 stabilized DAT in inward-occluded ad outward-open conformations, respectively.

We measured the volumes by counting the water molecules in the vestibules (see Methods) and found that hDAT/JJC8-089 (orange) has ~20.3 more waters in the extracellular vestibule (**A**) and ~6.4 fewer waters in the intracellular vestibule (**B**) than hDAT/JJC8-091 (blue).

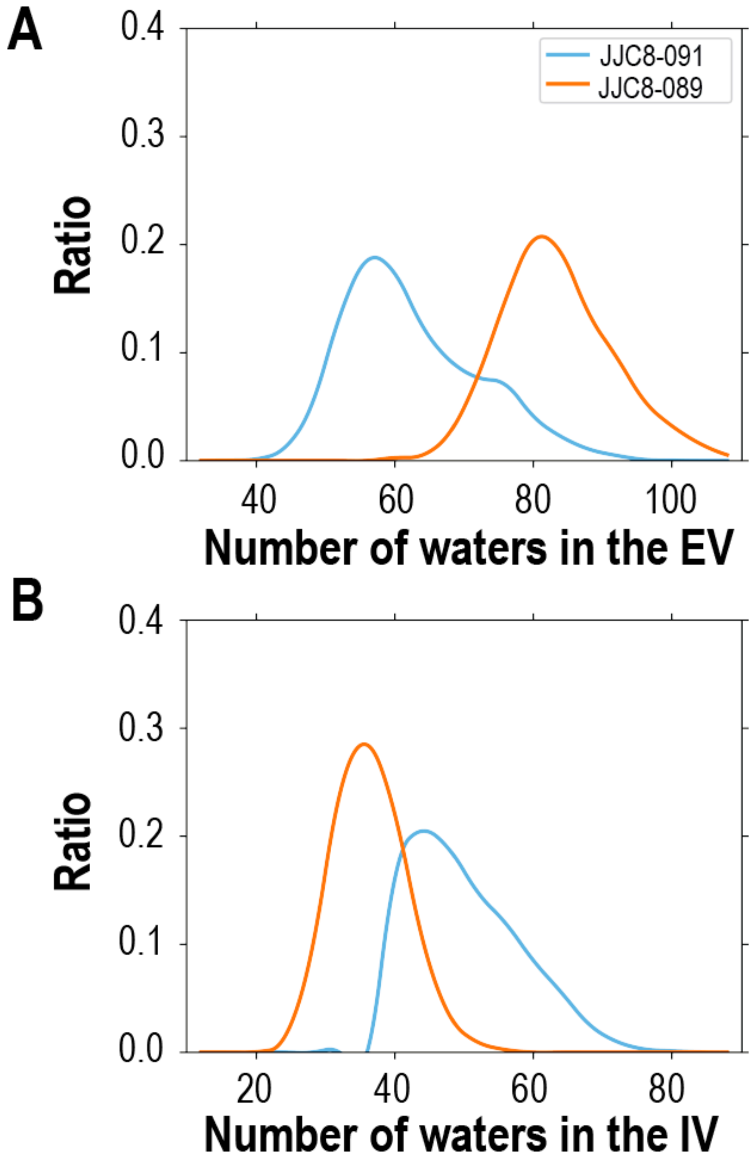

Figure S3. Distribution of ligand end to end distance.

Ligand end-to-end distance was calculated using two defined carbon atoms as shown in red dots (**A**). Distribution of ligand end-to-end distance of those carbons of hDAT/RDS04-010 and hDAT/RDS03-094 is shown in (**B**), and of hDAT/JJC8-091 and hDAT/JJC8-089 is shown in (**C**).

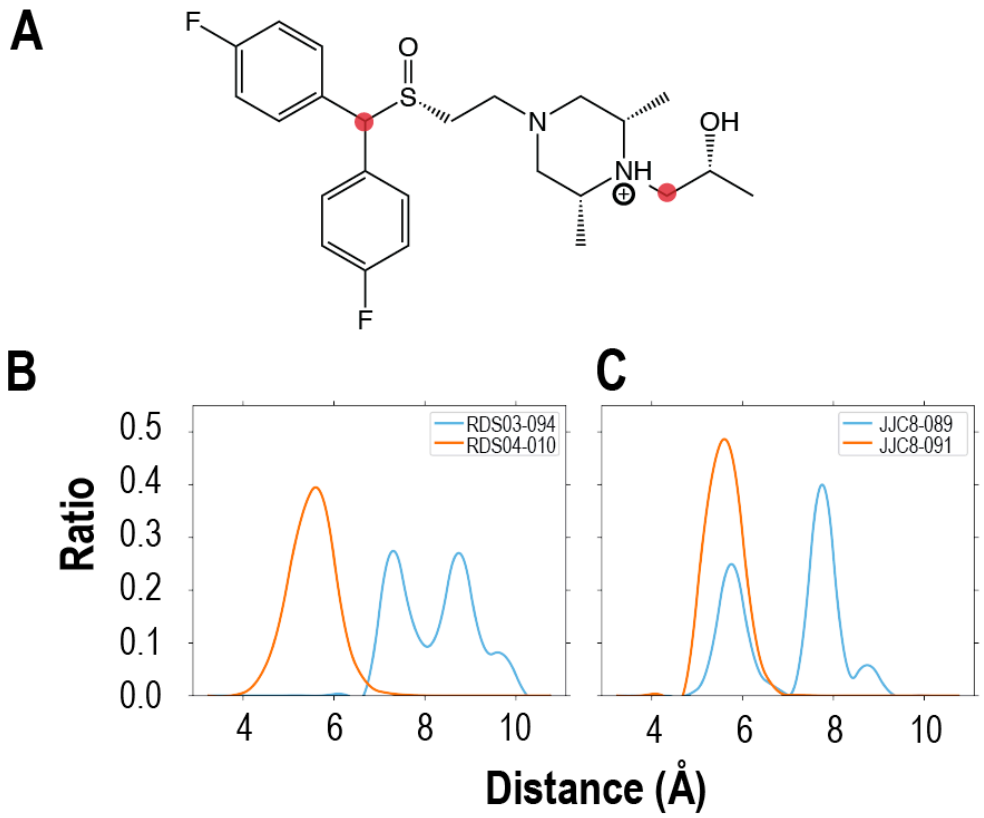

Figure S4. The sulfoxide substitution attracts more water than sulfide.

The difference between inward-facing and outward-facing ensembles was shown in terms of the number of water counts around the sulfur atom of substitution sulfoxide and sulfide (**A**) and the distribution of minimum water-sulfur atom distance (**B**). Upon counting the water molecules near the sulfur atom (within 3.5 Å), it was found that there are more water molecules near sulfoxide compared to sulfide (panel **A**). For substitution of sulfoxide, it's 59.8% for RDS04-010 and 50.9% for JJC8-091 that having more than one water molecule. For substitution of sulfide, it's 3.8% for RDS03-094 and 2.0% for JJC8-089. In panel B, the minimum distance between the closest water to the sulfur atom is used to compare the water nearby sulfoxide and sulfide substitution. Those water nearby sulfoxide can form a single peak around 3.1 Å and 3.3 Å for both RDS04-010 and JJC8-091, suggesting a direct hydrogen bond was formed between sulfoxide and water. For sulfide substitution, the peaks are around 4.7 Å and 5.1 Å for RDS03-094 and JJC8-089 respectively.

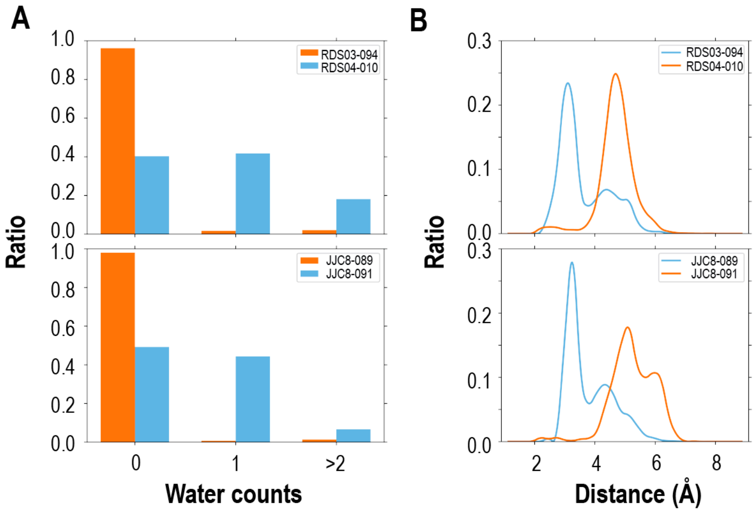

Figure S5. hDAT/JJC8-091 has a tendence to be more inward-facing compared to hDAT/JJC8-089.

Extracellular (**A**) and intracellular (**B**) PIA distance matrixes were shown more inward-facing (blueish color in panel **B**) for JJC8-091 and more outward-facing (reddish color in panel **A**) for JJC8-089. The superimposition of the equilibrated DAT/ JJC8-091(light blue) and DAT/ JJC8-089 (light pink) complexes show the inward movement of TM1e, EL3, and TM6e (**C**) and the outward movement of TM1i (**D**).

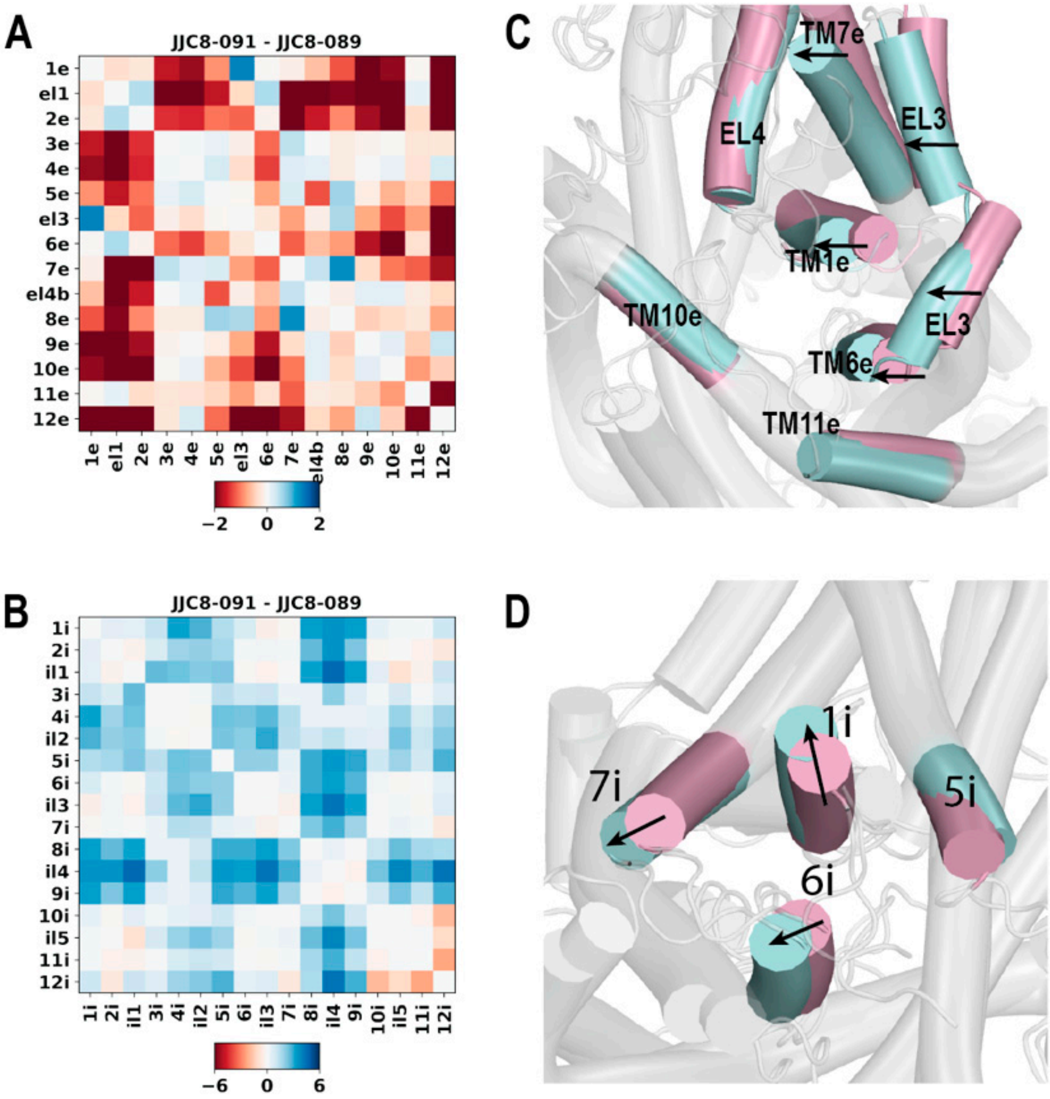

Figure S6. F320 χ1 sampled more trans conformation for compounds with substitution of sulfoxide.

The distribution of F320 χ1 angle for RDS04-010 and RDS03-094 (**A**), and for JJC8-091 and JJC8-089 (**B**).

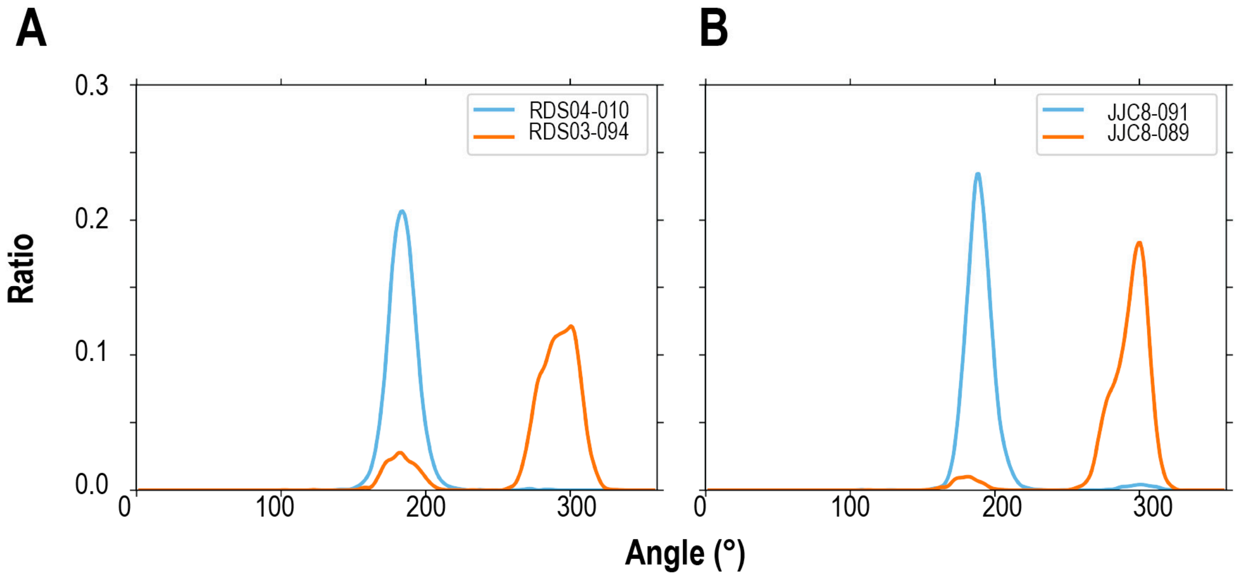

Movie S1. The transition to an inward-facing conformation observed in a representative hDAT/RDS04-010 MD simulation trajectory.

The intracellular portion of the TM1 (TM1i) and N-terminal loop are colored in magenta. Na ions are shown in purple spheres. The carbon atoms the bound RDS04-010 are in green. The dissociation of the Na2 leads to the swinging out the TM1 and detachment of the N-terminal loop from the TM domain, while RDS04-010 transitions to a more extended conformation in the newly created cavity by these rearrangements.
